# The Effectiveness of Vitamin D Supplementation in Association with Non-Surgical Periodontal Therapy: A Systematic Review

**DOI:** 10.3390/dj14040211

**Published:** 2026-04-06

**Authors:** Paolo Pesce, Francesco Bagnasco, Nicola de Angelis, Gaetano Isola, Cecilia Portaccio, Marco Migliorati, Maria Menini

**Affiliations:** 1 Department of Surgical Sciences (DISC), University of Genoa, 16148 Genova, Italy; 2 Unit of Periodontology, Department of General Surgery and Surgical-Medical Specialties, School of Dentistry, University of Catania, 95123 Catania, Italy; gaetano.isola@unict.it

**Keywords:** vitamin D, periodontitis, non-surgical periodontal therapy, scaling and root planing, supplementation, 25-hidroxyvitamin D, immune modulation

## Abstract

**Background**: Vitamin D has been increasingly investigated for its pleiotropic immunomodulatory and antimicrobial effects, which may influence periodontal inflammation and healing. This systematic review aimed to evaluate the impact of serum vitamin D levels and vitamin D supplementation as an adjunct to non-surgical periodontal therapy (NSPT) on clinical and microbiological outcomes in patients with periodontitis. **Methods**: An electronic search was conducted in MEDLINE and other major databases up to September 2025. Randomized controlled trials assessing the relationship between vitamin D status or supplementation and periodontal outcomes following NSPT were included. Data were synthesized qualitatively, focusing on changes in serum 25(OH)D levels and periodontal parameters, including probing pocket depth (PPD), clinical attachment level (CAL), plaque index (PI), and gingival inflammation. **Results**: Four studies met the inclusion criteria. In patients with sufficient baseline vitamin D levels, supplementation provided limited additional clinical benefits beyond NSPT alone. Conversely, in vitamin D-deficient patients, supplementation regimens capable of restoring serum 25(OH)D levels above 30 ng/mL were consistently associated with greater reductions in PPD, improved CAL, and decreased plaque and bleeding indices. Microbiological analyses also revealed a reduction in red complex periodontal pathogens in supplemented groups. **Conclusions**: Vitamin D supplementation enhances the clinical effectiveness of NSPT primarily in patients with documented vitamin D deficiency. Its adjunctive benefits appear to be mediated by immunomodulatory and antimicrobial mechanisms that complement mechanical debridement. While current evidence supports targeted supplementation in deficient individuals, long-term randomized trials are required to establish standardized protocols and confirm sustained clinical benefits.

## 1. Introduction

Periodontitis is a chronic inflammatory condition that affects the supporting structures of the teeth and is driven by multiple interacting factors. It is characterized by a gradual and often irreversible breakdown of periodontal tissues—gingiva, periodontal ligament, cementum, and alveolar bone—with clinical manifestations such as clinical attachment loss (CAL), radiographically detectable bone loss (RBL), periodontal pocket formation, bleeding on probing, and, in more advanced stages, tooth mobility and eventual tooth loss [1,2]. Globally, it remains one of the main causes of tooth loss in adulthood. From a biological standpoint, periodontitis originates from a dysbiotic microbial community capable of triggering an exaggerated host immune response. The disease is therefore considered multifactorial; its onset and progression depend not only on the virulence of oral pathogens and their interactions but also on host-related factors, including genetic predisposition, individual susceptibility, and lifestyle or environmental variables such as smoking habits, diet, and systemic health conditions [3]. In recent years, considerable scientific attention has been devoted to understanding how systemic and nutritional factors influence the host response to periodontal infection. Among these, vitamin D has gained relevance. While traditionally recognized for its essential role in calcium balance and skeletal metabolism, vitamin D is now acknowledged to exert important immunomodulatory functions, affecting both innate and adaptive immune pathways [4,5]. Its active form, 1,25-dihydroxyvitamin D_3_ (calcitriol), binds to the vitamin D receptor (VDR), which is expressed in several immune cell types and in periodontal tissues, regulating the transcription of numerous genes involved in inflammatory processes, antimicrobial defense, and bone remodeling [6]. This biological background supports the hypothesis that inadequate vitamin D levels may heighten the risk of developing periodontal disease by compromising bone homeostasis and altering the host’s immune competence against oral microorganisms [7,8,9,10]. Epidemiological studies reinforce this association: multiple cross-sectional and longitudinal investigations have reported an inverse relationship between serum concentrations of 25(OH)D and both the prevalence and the severity of periodontitis [11,12]. Findings from the National Health and Nutrition Examination Survey (NHANES) demonstrated that individuals with higher vitamin D levels present reduced attachment loss and shallower periodontal pockets, even when adjusting for major confounders such as smoking status and diabetes [13]. Mechanistically, vitamin D sustains periodontal integrity through several biological actions. It enhances the synthesis of antimicrobial peptides—such as cathelicidin (LL-37) and β-defensins—which act against bacterial, viral, and fungal components of the subgingival microbiota [14]. It modulates the activity of monocytes and macrophages, promoting a less pro-inflammatory phenotype and supporting the resolution of inflammation [10]. Additionally, vitamin D downregulates Th1 and Th17 responses—key sources of IFN-γ and IL-17—and promotes regulatory T cells (Treg), which are essential in containing immune-mediated tissue destruction [15].

Several clinical trials have examined whether vitamin D supplementation may enhance the outcomes of non-surgical periodontal therapy (NSPT), primarily scaling and root planing (SRP). Many of these studies have found that patients who receive vitamin D alongside SRP experience greater improvements in probing depth reduction and clinical attachment gain compared with those treated with SRP alone [16,17,18]. Other trials have reported reductions in bleeding on probing, plaque accumulation, and local or systemic inflammatory mediators such as IL-6 and TNF-α in serum or gingival crevicular fluid [19]. Notably, these effects appear more evident in individuals presenting baseline vitamin D deficiency, typically defined as serum levels below 30 ng/mL, suggesting that restoring physiological concentrations enhances tissue healing and immune regulation [20]. This evidence supports the incorporation of vitamin D status assessment into the periodontal diagnostic process and the consideration of supplementation when deficiencies are detected. Despite these observations, results across the literature remain partly inconsistent. Some studies report minimal or non-significant improvements, due to differences in supplementation protocols, duration of treatment, initial vitamin D status, formulations used, or patient characteristics such as skin phototype, body mass index, or medical comorbidities [21]. Meta-analyses confirm a beneficial trend but highlight substantial heterogeneity and emphasize the need for large, well-designed randomized clinical trials to clarify optimal dosage strategies [11]. Clinically, the addition of vitamin D to periodontal treatment appears safe, inexpensive, and potentially advantageous. When administered at appropriate doses, vitamin D presents low toxicity and offers systemic benefits beyond periodontal health, such as improved skeletal integrity and reduced susceptibility to autoimmune or infectious diseases [22]. In conclusion, current data indicate that vitamin D plays a meaningful role in modulating the immune response and supporting periodontal tissue stability. Supplementation, particularly in deficient patients, represents a promising adjunct to conventional non-surgical therapy. However, further high-quality randomized studies are required to define standardized protocols and to validate long-term clinical benefits, although the biological rationale and existing evidence strongly support its potential contribution to periodontal care.

## 2. Materials and Methods

### 2.1. Protocol and Registration

This systematic review was conducted following the Preferred Reporting Items for Systematic Reviews and Meta-Analyses (PRISMA 2020) guidelines [23,24] (File S1). The review protocol was registered prospectively in the International Prospective Register of Systematic Reviews (PROSPERO) under the identification number [CRD420251062545].

### 2.2. Focused Questions and Eligibility Criteria

A focused question was created according to the PICO format: Can vitamin D supplementation improve the effectiveness of non-surgical periodontal therapy? The PICO format comprises the following elements:

- Population (P): patients affected by periodontitis.
- Intervention (I): patients who underwent non-surgical therapy and received vitamin D supplementation.
- Comparison (C): patients who underwent non-surgical therapy and received no supplementation or placebo.
- Outcome (O): to evaluate the differences in periodontal parameters. Primary: PPD Secondary: CAL, BOP, and PI.

For the development of the present systematic review, only studies that met the following inclusion criteria were considered:

(1) Randomized clinical trials
(2) Human studies

Conversely, articles were excluded if they met any of the following criteria:

1. Controlled clinical trials
2. Prospective non-randomized studies
3. Case–control studies
4. Systematic reviews
5. Meta-analyses
6. Case reports
7. Animal studies
8. In vitro studies
9. Lack of full-text availability

### 2.3. Search Strategy and Data Extraction

Electronic research was performed on three databases: Medline (PubMed), Scopus, and the Cochrane Central Register of Controlled Clinical Trials (CENTRAL). The last search was conducted in September 2025. The following search strategy was used on PUBMED and adapted for each database: ((periodontitis) and (treatment or therapy or surgical or “non-surgical”)) and (vitamin d). Additionally, the reference lists of all included articles, as well as those of relevant systematic reviews, were manually examined to identify any further eligible studies that may have been missed during the electronic search. No restrictions were applied regarding the date of publication, but only articles written in English were selected. Two authors (CP and PP) screened the titles, abstracts, and full texts. The complete texts of all studies deemed potentially suitable were retrieved for detailed assessment, and any article subsequently excluded was removed with a clearly documented justification. The data extraction process was performed by the same two authors (CP and PP) using a Microsoft Excel spreadsheet. The extracted information included the year of publication, authors, study title, study design, number of patients and mean age, type of treatment, and the reported outcomes.

### 2.4. Risk of Bias in Individual Studies

Two authors (CP and PP) independently assessed the risk of bias in the included studies. The Cochrane Risk of Bias Tool (RoB 2.0) was used. Any differences in judgment were resolved through mutual discussion, with the involvement of a third reviewer when agreement could not be reached.

### 2.5. Synthesis Methods

The synthesis of results was conducted following a structured comparative approach. Due to heterogeneity in study design, vitamin D supplementation regimens, baseline serum 25(OH)D levels, and variability in reported periodontal outcomes, a quantitative meta-analysis could not be reliably performed. Instead, a qualitative synthesis was undertaken. Clinical outcomes—including probing depth (PD), clinical attachment level (CAL), bleeding on probing (BOP), and plaque indices (PI)—were extracted from each study along with serum 25(OH)D concentrations when available. Studies were grouped according to the vitamin D status of participants at baseline (deficient vs. non-deficient) to allow for targeted comparison of treatment effects. When available, microbiological and inflammatory biomarkers were also considered to support the mechanistic interpretation of clinical changes. Where the data allowed for comparison, differences in mean change from baseline between intervention and control groups were examined. The consistency and directionality of treatment effects were prioritized over absolute effect size due to variability in measurement protocols and follow-up duration. Results were therefore interpreted by integrating clinical outcomes, biological plausibility, and the strength of the study methodology.

## 3. Results

### 3.1. Study Selection

**Figure 1:**
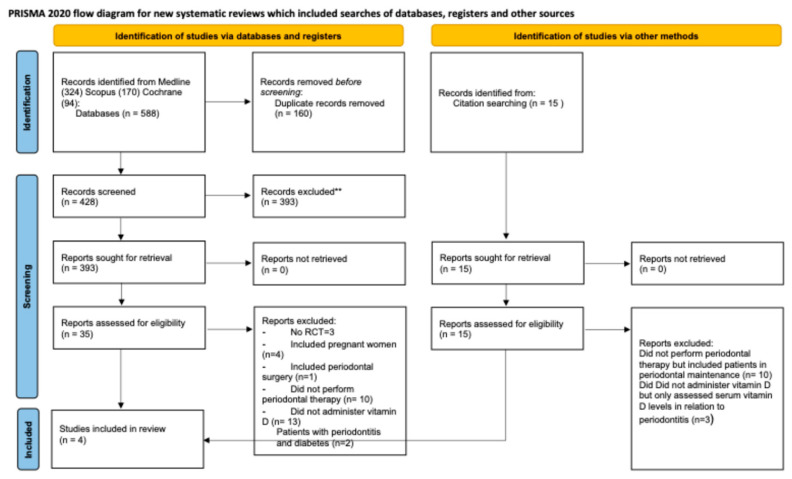
Flowchart of the included studies.

Exploring online databases led to 588 articles, plus 15 additional records identified through references in systematic reviews, for a total of 603 studies. Four studies were included in the systematic review. The selection process is visually represented in Figure 1.

### 3.2. Studies Characteristics

The four studies included in this systematic review evaluated the effects of vitamin D supplementation as an adjunct to non-surgical periodontal therapy. Two of the included studies enrolled patients who were vitamin D deficient at baseline [18,19], whereas two studies involved subjects with normal serum vitamin D levels [21,25]. The main data are reported in Table 1.

Gao et al. (2020) [21] included patients with moderate-to-severe periodontitis without requiring baseline vitamin D deficiency. Participants received non-surgical periodontal therapy and were then randomized to vitamin D3 (1000 IU/day or 2000 IU/day) or placebo for three months. Supplementation led to a dose-dependent increase in serum 25(OH)D levels and small but statistically significant improvements in probing depth and clinical attachment, although clinical differences were modest (0.1–0.3 mm).

Meghil et al. (2019) [25] also investigated patients without confirmed vitamin D deficiency, focusing instead on individuals with darker skin phototypes, who are predisposed to lower vitamin D synthesis. Supplementation with 4000 IU/day for 16 weeks modulated systemic immune parameters, particularly reducing CD3+ and CD8+ cytotoxic T-cell counts following SRP. The results suggested that vitamin D may mitigate immune overactivation even in patients who are not overtly deficient.

Perić et al. (2020) [18] included vitamin D-deficient subjects and administered weekly 25,000 IU of vitamin D3 for six months. Supplementation successfully normalized serum vitamin D levels and produced a trend toward greater reduction of probing depths ≥ 5 mm compared to placebo, though intergroup differences did not reach statistical significance, due to the limited sample size.

Assaf and Aboelsaad (2019) [19] included patients with chronic periodontitis and documented vitamin D deficiency. Supplementation with 10,000 IU/day for 12 weeks produced markedly greater improvements in plaque, gingival inflammation, probing depth, and attachment levels compared to placebo, demonstrating a clear clinical benefit in reversing deficiency.

### 3.3. Risk of Bias

**Figure 2:**
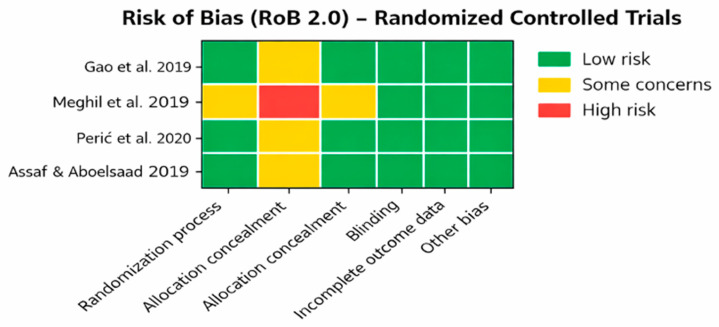
Risk of Bias. (Gao et al. 2020 [21], Meghil et al. 2019 [25], Perić et al. 2020 [18], Assaf and Aboelsaad 2019 [19]).

The four randomized controlled trials were evaluated using the Cochrane RoB 2.0 tool. Overall, the randomized trials demonstrated adequate methodological quality, with generally low risk of bias in the randomization process and completeness of outcome data. However, in several studies, allocation concealment procedures and examiner blinding were insufficiently reported, contributing to an overall classification of some concerns in these domains. Despite these limitations, no study was judged to have a level of bias high enough to warrant exclusion from the review, and all were considered to provide data of sufficient quality to contribute to the qualitative synthesis. The results of the risk of bias analysis are reported in Table 2 and Figure 2.

### 3.4. Synthesis of Result

Two studies enrolled patients with documented vitamin D deficiency (serum 25(OH)D < 30 ng/mL), while two studies did not control baseline vitamin D status. Across all studies, non-surgical periodontal therapy (NSPT), mainly scaling and root planing (SRP), resulted in significant clinical improvements; however, greater benefits were observed when vitamin D supplementation was administered to deficient individuals.

In the two studies involving vitamin D-deficient patients (Perić 2020 [18]; Assaf and Aboelsaad 2019 [19]), supplementation restored serum 25(OH)D levels to ≥30 ng/mL and was associated with enhanced probing pocket depth (PPD) reduction and greater clinical attachment level (CAL) gains compared to SRP alone. The additional PPD reduction attributable to vitamin D ranged from 0.3 to 1.0 mm, while CAL gains ranged from 0.3 to 0.8 mm, depending on disease severity and dosage. Notably, Assaf and Aboelsaad reported a 45% reduction in mean PPD in the supplemented group versus 18.9% in controls over 12 weeks, with a 32.7% greater CAL improvement. Perić et al. reported that supplementation with 25,000 IU/week for six months normalized serum vitamin D levels and reduced deep residual pockets (≥4–5 mm) more than placebo, although differences were not statistically significant, likely due to the limited sample size.

Conversely, studies conducted in populations without confirmed vitamin D deficiency (Gao et al. 2020 [21]; Meghil 2019 [25]) reported modest clinical benefits. Gao et al. observed small but statistically significant differences in PPD and CAL reduction (0.1–0.3 mm), while Meghil et al. reported changes mainly in inflammatory markers and T-cell modulation rather than clear clinical periodontal improvements. Overall, these findings indicate that vitamin D supplementation provides a clinically meaningful adjunctive benefit to NSPT primarily in deficient patients, supporting targeted assessment and supplementation rather than routine universal use.

The main results are reported in Table 3.

## 4. Discussion

The findings of this systematic review highlight marked variability in the clinical effectiveness of vitamin D supplementation as an adjunct to non-surgical periodontal therapy (NSPT), largely dependent on baseline vitamin D status. Studies conducted in periodontally diseased patients with sufficient serum 25(OH)D levels [15,21]) demonstrated clear biological activity of vitamin D but only modest and clinically negligible benefits. Both studies confirmed the pleiotropic immunomodulatory role of vitamin D, including the modulation of cytotoxic T-cell activity, reduction in pro-inflammatory cytokines, stimulation of antimicrobial peptides, and activation of autophagic pathways. Meghil et al. (2019) [25] reported increased serum 25(OH)D levels, reductions in CD3+ and CD8+ T-cells, decreased salivary IL-1β, TNF-α, and IL-6, and increased expression of autophagy-related proteins (ATG5, ATG7, ATG16L1, Beclin-1); however, these biological changes did not translate into superior clinical improvements in CAL, PD, or BOP beyond those achieved by SRP alone. Similarly, Gao et al. (2020) [21], in a large randomized controlled trial (360), observed statistically significant but clinically minimal improvements in PD and attachment levels (0.1–0.3 mm) following supplementation with 1000–2000 IU/day for three months, with no significant effects on bleeding or plaque indices.

In contrast, studies involving vitamin D-deficient populations consistently demonstrated more pronounced clinical benefits. Randomized trials and controlled studies that successfully restored serum 25(OH)D levels above the sufficiency threshold (>30 ng/mL) reported greater reductions in probing depth, more substantial clinical attachment gains, and significant decreases in plaque and bleeding indices compared with SRP alone. In the trial by Assaf and Aboelsaad (2019) [19], supplementation with 10,000 IU/day (five days per week) for 12 weeks increased mean serum 25(OH)D from 14.36 to 37.64 ng/mL and resulted in marked improvements, with PD reduced by 45% versus 18.9% and CAL improved by 32.7% versus 19.5% in the placebo group. Comparable trends were observed in the studies by Perić et al. (2020) [18] and Govindharajulu et al. (2024) [26], the latter uniquely demonstrating a significant reduction in red-complex pathogens (Porphyromonas gingivalis, Tannerella forsythia, Treponema denticola), supporting a modulatory effect of vitamin D on the subgingival microbiota, potentially mediated by antimicrobial peptides such as LL-37. The non-randomized study by Kaur et al. (2024) [20] further confirmed clinically relevant improvements following high-dose repletion in vitamin D-deficient women, while epidemiological data from Thanoon and Al-Mashhadane (2023) [17] reinforced the association between low serum 25(OH)D levels and increased periodontal severity. Overall, the evidence indicates that vitamin D supplementation provides a meaningful adjunctive benefit to NSPT only when three conditions are met: documented vitamin D deficiency, an adequate supplementation regimen capable of restoring serum sufficiency (typically 50,000–60,000 IU/week or 10,000 IU/day), and combination with SRP rather than use as a standalone intervention. Under these circumstances, improvements in periodontal inflammation, microbial profile, and clinical outcomes are consistently greater than those achieved with mechanical therapy alone.

### 4.1. Clinical Implications

The findings of this systematic review suggest that vitamin D assessment should be considered during periodontal evaluation, particularly in patients presenting with moderate to severe periodontitis or with known systemic risk factors. In individuals with documented vitamin D deficiency, supplementation appears to enhance the clinical effectiveness of non-surgical periodontal therapy by improving probing depth reduction, clinical attachment gain, and inflammatory control [27]. When adequately dosed and combined with scaling and root planing, vitamin D supplementation represents a safe, low-cost, and biologically plausible adjunctive strategy, potentially contributing to improved short-term periodontal outcomes. However, routine supplementation in vitamin D-sufficient patients cannot currently be recommended based on available evidence.

### 4.2. Limitations and Future Recommendations

Although a formal GRADE assessment based on pooled effect estimates would have been desirable, this was not feasible in the present review because a meta-analysis could not be performed due to substantial heterogeneity in outcome reporting and incomplete quantitative data across the included randomized controlled trials. Therefore, the certainty of the evidence should be interpreted with caution, as it is limited by inconsistency in reported clinical outcomes, small sample sizes, and differences in data presentation among studies. However, based on the current evidence, the strength of recommendation for vitamin D supplementation as an adjunct to non-surgical periodontal therapy can be considered moderate. Clinical trials and controlled studies suggest that vitamin D supplementation may enhance the outcomes of scaling and root planing, particularly in patients with documented vitamin D deficiency. In these subjects, adjunctive supplementation is associated with greater reductions in probing pocket depth, improvements in clinical attachment level, and a decrease in inflammatory periodontal parameters compared with mechanical therapy alone. Conversely, in patients with sufficient baseline vitamin D levels, the additional clinical benefit appears limited and of modest magnitude, despite evidence of biological and immunomodulatory activity. The heterogeneity of study designs, supplementation regimens, baseline serum levels, and follow-up periods, together with the predominantly short-term nature of the available data, limits the strength of universal clinical recommendations. Therefore, vitamin D supplementation may be recommended as an adjunctive strategy in vitamin D-deficient periodontal patients undergoing non-surgical periodontal therapy, while routine supplementation in all periodontal patients cannot yet be supported. Further well-designed, long-term randomized clinical trials are required to establish standardized protocols and to confirm the stability of clinical benefits over time.

A further limitation of the present review is that the search strategy, although structured across the main databases, was not fully optimized with database-specific controlled vocabulary (e.g., MeSH terms in PubMed) and an expanded set of alternative keywords/synonyms, such as ‘periodontal disease’, ‘scaling and root planing’, ‘SRP’, ‘25-hydroxyvitamin D’, and ‘vitamin-D’; therefore, some potentially relevant studies may not have been identified

## 5. Conclusions

Vitamin D supplementation appears to be a clinically relevant adjunct to non-surgical periodontal therapy in patients with documented vitamin D deficiency, enhancing periodontal healing through immunomodulatory and antimicrobial mechanisms. While biologically active in vitamin D-sufficient individuals, its additional clinical benefit in this population remains limited. Current evidence supports targeted supplementation rather than routine use. Further well-designed clinical trials are required to confirm long-term efficacy and to establish standardized supplementation protocols in periodontal care.

## Figures and Tables

**Table 1:** Main data for the included articles.

Author and Year	Type of Study	Numbers of Patients	Test Therapy	Control Therapy	Follow Up	Parameters Evaluated
Weimin Gao et al. 2020 [21]	RCT	360	Supragingival and subgingival scaling and root planing (SRP) followed by oral vitamin D_3_ supplementation (1000 or 2000 IU/day for 3 months)	SRP followed by placebo capsules	J 3 months	K Serum 25(OH)D, PD, AL, BI, PLI, ACH
Mohamed M. Meghil 2019 [25]	RCT	23	Non-surgical periodontal therapy (SRP) plus oral vitamin D_3_ (4000 IU/day for 16 weeks)	Non-surgical periodontal therapy (SRP) plus placebo	3.5 months	Serum vitamin D levels, CAL, PD, BOP and GI
Marina Perić et al. 2020 [18]	RCT	26	SRP performed in two sessions within 48 h plus vitamin D_3_ supplementation (25,000 IU/week), starting 1 month before therapy and continued for 6 months	SRP performed in two sessions within 48 h plus weekly placebo	6 months	Vitamin D_3_ Serum Levels, FMBS (Full Mouth Bleeding Score), FMPS (Full Mouth Plaque Score) and PPD
Assaf and Aboelsaad 2019 [19]	RCT	29	SRP combined with oral vitamin D_3_ (10,000 IU/day, 5 days/week for 12 weeks)	SRP combined with placebo	3 months	Vitamin D Blood Level, GI, PI, PPD and CAL

**Table 2:** Risk of bias.

Study	Design	Tool	Randomization	Blinding	Outcome Data	Overall RoB
Gao et al., 2020 [21]	RCT	RoB 2.0	Low	Unclear	Low	Some concerns
Meghil et al., 2019 [25]	RCT	RoB 2.0	Low	Low	Low	Low
Perić et al., 2020 [18]	RCT	RoB 2.0	Some concerns	Low	Low	Some concerns
Assaf and Aboelsaad 2019 [19]	RCT	RoB 2.0	Low	Unclear	Low	Some concerns

**Table 3:** Main results of included studies. PPD = Probing Pocket Depth; CAL = Clinical Attachment Level; PI = Plaque Index; GI = Gingival Index. Values are expressed as mean standard deviation (SD). PRE CTR = Group control pre-treatment. POST CTR = Group control post-treatment. PRE TEST = Group test pre-treatment. POST TEST = Group control post-treatment. * Number of sites > 4 mm.

	PPD (Mean ± SD)				CAL (Mean ± SD)				GI (Mean ± SD)				PI (Mean ± SD)			
	PRE CTR	POST CTR	PRE TEST	POST TEST	PRE CTR	POST CTR	PRE TEST	POST TEST	PRE CTR	POST CTR	PRE TEST	POST TEST	PRE CTR	POST CTR	PRE TEST	POST TEST
**Gao et al., 2020 [21]**	2.5 ± 0.5	2.4 ± 0.5	2.6 ± 0.5	2.5 ± 0.4	3.5 ± 0.7	3.2 ± 0.6	3.6 ± 0.6	3.4 ± 0.7	1.5 ± 0.8	0.9 ± 0.3	1.6 ± 0.8	1.1 ± 0.6	1.8 ± 0.9	1.6 ± 0.7	1.9 ± 0.8	1.7 ± 0.8
**Assaf and Aboelsaad 2019 [19]**	3.199	3.009	2.271	1.821	2.308	2.113	1.925	1.598	0.954	0.8064	1.182	0.849	1.063	0.868	0.881	0.587
**Perić et al., 2020 [18]**	49.64 *	20.45 *	67.70 *	24.80 *									0.55 ± 0.37	0.23 ± 0.17	0.45 ± 0.22	0.29 ± 0.19

## Data Availability

The original contributions presented in this study are included in the article/Supplementary Materials. Further inquiries can be directed to the corresponding authors.

## Supplementary Materials

The following supporting information can be downloaded at https://www.mdpi.com/article/10.3390/dj14040211/s1, File S1: PRISMA 2020 Checklist.

## References

[1] Kwon T., Lamster I.B., Levin L. (2021). Current concepts in the management of periodontal disease. Int. Dent. J..

[2] Chmielewski M., Pilloni A., Cuozzo A., D’albis G., D’elia G., Papi P., Marini L. (2025). The 2018 Classification of Periodontitis: Challenges from Clinical Perspective. Dent. J..

[3] Hajishengallis G., Chavakis T. (2021). Local and systemic mechanisms linking periodontal disease and inflammatory comorbidities. Nat. Rev. Immunol..

[4] Martens P.-J., Gysemans C., Verstuyf A., Mathieu C. (2020). Vitamin D’s Effect on Immune Function. Nutrients.

[5] Aranow C. (2020). Vitamin D and the immune system. J. Investig. Med..

[6] Botelho J., Machado V., Proença L., Delgado A.S., Mendes J.J. (2020). Vitamin D Deficiency and Oral Health: A Comprehensive Review. Nutrients.

[7] Li Y., Wang J., Cai Y., Chen H. (2023). Association of Serum Vitamin D With Periodontal Disease. Int. Dent. J..

[8] Pradhan S., Agrawal S. (2021). Serum Vitamin D in Patients with Chronic Periodontitis and Healthy Periodontium. J. Nepal Health Res. Counc..

[9] Al Anazi Y., Almutairi M., Alswayyed A., Alshammari A., Alshammari N., Alqahtani A., Nazir M.A. (2023). Association of Serum Vitamin D with Periodontal Disease. Int. Dent. J..

[10] Liu K., Meng H. (2023). The role of vitamin D in periodontal health and disease. J. Periodontal Res..

[11] Ramaprabha G., Khan N.S., Kunusoth R., Kakati I., Qadri S.S.H., Seshadri P.R. (2023). Assessment of outcome of oral supplementation of Vitamin D3 as an adjunct to scaling and root planing in chronic periodontitis patients with type II diabetes mellitus: A randomized controlled clinical trial. J. Pharm. Bioallied Sci..

[12] Liang F., Zhou Y., Zhang Z., Zhang Z., Shen J. (2023). Association of vitamin D in individuals with periodontitis: An updated systematic review and meta-analysis. BMC Oral Health.

[13] Dragonas P., El-Sioufi I., Bobetsis Y.A., Madianos P.N. (2020). Association of Vitamin D With Periodontal Disease: A Narrative Review. Oral Health Prev. Dent..

[14] Gombart A.F., Pierre A., Maggini S. (2020). A Review of Micronutrients and the Immune System-Working in Harmony to Reduce the Risk of Infection. Nutrients.

[15] Grant W.B., van Amerongen B.M., Boucher B.J. (2023). Periodontal Disease and Other Adverse Health Outcomes Share Risk Factors, including Dietary Factors and Vitamin D Status. Nutrients.

[16] Machado V., Lobo S., Proença L., Mendes J.J., Botelho J. (2020). Vitamin D and Periodontitis: A Systematic Review and Meta-Analysis. Nutrients.

[17] Thanoon A., Al-Mashhadane F. (2023). Relationship Between Vitamin D Deficiency and Chronic Periodontitis. Georgian Med. News.

[18] Perić M., Maiter D., Cavalier E., Lasserre J.F., Toma S. (2020). The Effects of 6-Month Vitamin D Supplementation during the Non-Surgical Treatment of Periodontitis in Vitamin-D-Deficient Patients: A Randomized Double-Blind Placebo-Controlled Study. Nutrients.

[19] Assaf M., Aboelsaad N. (2019). The Effectiveness of Vitamin D Supplementation in Chronic Periodontitis Patients: A Randomized Controlled Clinical Trial. Egypt. Dent. J..

[20] Kaur J., Kaur S., Sarangal V., Narang R.D.S., Singh S.T., Khindri D. (2024). To Evaluate the Association Between Serum Concentration of Vitamin D and Chronic Periodontitis in Non-menopausal Females: A Clinico Biochemical Study. Curr. Drug Saf..

[21] Gao W., Tang H., Wang D., Zhou X., Song Y., Wang Z. (2020). Effect of short-term vitamin D supplementation after nonsurgical periodontal treatment: A randomized, double-masked, placebo-controlled clinical trial. J. Periodontal Res..

[22] Isola G. (2020). The Impact of Diet, Nutrition and Nutraceuticals on Oral and Periodontal Health. Nutrients.

[23] Moher D., Liberati A., Tetzlaff J., Altman D.G., Group P. (2009). Preferred reporting items for systematic reviews and meta-analyses: The PRISMA statement. PLoS Med..

[24] Page M.J., McKenzie J.E., Bossuyt P.M., Boutron I., Hoffmann T.C., Mulrow C.D., Shamseer L., Tetzlaff J.M., Akl E.A., Brennan S.E. (2021). The PRISMA 2020 statement: An updated guideline for reporting systematic reviews. BMJ.

[25] Meghil M.M., Hutchens L., Raed A., Multani N.A., Rajendran M., Zhu H., Looney S., Elashiry M., Arce R.M., Peacock M.E. (2019). The influence of vitamin D supplementation on local and systemic inflammatory markers in periodontitis patients: A pilot study. Oral Dis..

[26] Govindharajulu R., Seshadri P.R., Syed N.K., Sukumaran B., Mathivanan S., Ramkumar N. (2024). Assessment of the Antibacterial Effect of Vitamin D3 against Red Complex Periodontal Pathogens: A Microbiological Assay. J. Contemp. Dent. Pract..

[27] Shah M., Poojari M., Kakkad D., Dutta S.B., Sinha S., Chowdhury K., Dagli N., Haque M., Kumar S., Poojary M.E. (2023). Vitamin D and Periodontal Health: A Systematic Review. Cureus.

